# A randomized controlled study on the efficacy of a novel combination vaccine against enzootic pneumonia (*Mycoplasma hyopneumoniae*) and porcine Circovirus type 2 (PCV2) in the presence of strong maternally derived PCV2 immunity in pigs

**DOI:** 10.1186/s12917-017-1014-7

**Published:** 2017-04-07

**Authors:** Panagiotis D. Tassis, Ioannis Tsakmakidis, Vassileios G. Papatsiros, Dimitrios Koulialis, Tom Nell, Georgia Brellou, Eleni D. Tzika

**Affiliations:** 1 Farm Animals Clinic, School of Veterinary Medicine, Aristotle University of Thessaloniki, St. Voutyra 11, 54627 Thessaloniki, Greece; 2 Clinic of Medicine, Faculty of Veterinary Science, University of Thessaly, P.O. Box 199, Trikalon 224, Karditsa, Greece; 3 Animal Health, Clinical Study Team Biologicals, P.O. Box 31, Boxmeer, 5830 AA the Netherlands; 4 Laboratory of Pathology, School of Veterinary Medicine, Aristotle University of Thessaloniki, University Campus, 54124 Thessaloniki, Greece

**Keywords:** Porcilis PCV M Hyo, Enzootic pneumonia, *Mycoplasma hyopneumoniae*, Porcine circovirus type 2, Pigs

## Abstract

**Background:**

*Mycoplasma hyopneumoniae* (M. hyo) and Porcine Circovirus Type 2 (PCV2) are major pathogens that cause significant health problems in swine worldwide. Maternal derived immunity (MDI) has been suggested as a significant immediate defence factor for newborn piglets and may interfere with piglet’s vaccination-induced immunity. The study aimed to assess the efficacy of a novel combination vaccine (consisting of PCV2 subunits and inactivated M. hyo strain J), against PCV2 and M. hyo natural infection [Porcilis^®^ PCV M Hyo (MSD Animal Health, Boxmeer, the Netherlands)], in the presence of strong maternally derived PCV2 immunity (antibody titre averaged 11.08 log_2_), under field conditions. The study was performed according to a controlled, randomized and blinded design in a Greek swine unit with Enzootic Pneumonia (EP) and subclinical PCV2 infection. In total, 600 healthy three-week-old suckling piglets were allocated randomly, either to treatment (vaccinated with the test product) or control group (injected with sterile buffered saline).

**Results:**

Vaccination significantly reduced the severity of lung lesions at slaughter (lesions of cranio-ventral pulmonary consolidation) (*P* < 0.001). The overall mean lung lesion score (LLS) was 9.6 in the vaccinated group and 12.2 in controls. The level of PCV2 viraemia was significantly reduced in vaccinated pigs. Furthermore, 25 g higher average daily weight gain (ADWG) was observed during the finishing phase (*P* < 0.001) and 18 g greater ADWG overall (*P* < 0.001).

**Conclusions:**

Results of LLS, PCV2 viremia and ADWG support the test product’s efficacy in the face of strong maternally derived PCV2 immunity.

## Background

*Mycoplasma hyopneumoniae* (M. hyo) and Porcine Circovirus Type 2 (PCV-2) are two major pathogens of ubiquitous nature that cause critical health disorders in swine worldwide. M. hyo is part of the aetiology of a chronic insidious bronchopneumonia, also described as enzootic pneumonia (EP) which causes severe economic losses in the global pig industry [1]. Other bacterial agents usually coexist in cases of EP along with M. hyo. The EP clinical manifestations include a dry, non-productive cough, variable reduction of feed intake and growth retardation [2]. Moreover, viral pathogens such as Porcine Reproductive and Respiratory Syndrome virus (PRRSv) and PCV2 have been suggested as agents that contribute further in converting EP into the more complex Porcine Respiratory Disease Complex [2, 3].

PCV-2, with four genotypes documented up to today (PCV2a, PCV2b, PCV2c, PCV2d-mPCV2b), has been etiologically associated with certain Porcine Circovirus Diseases (PCVD) [4]. Those include disorders such as PCV2 - Systemic Disease (PCV2-SD) (formerly known as postweaning multisystemic wasting syndrome) which is characterised by growth retardation - wasting, anaemia, possible respiratory problems and diarrhoea, as well as lymphocyte depletion and immune suppression. Moreover, PCV2 is present in the aetiology of porcine dermatitis and nephropathy syndrome, and clinical manifestations of pneumonia, reproductive failure or intestinal disorders [5–7]. However, subclinical PCV2 infection occurs frequently and although clinically “silent”, gradually results in significant economic losses due to growth retardation [8].

Vaccination of piglets is the main control method against clinical disease associated with M. hyo (eg. more than 85% of US farms vaccinate against M. hyo) [1, 3]. For almost a decade (since 2007), control of PCVD has been also based on vaccination of sows and/or piglets [3, 9]. Nevertheless, those two pathogens persist, circulate and interact with other pathogens in the host, making their clearance extremely difficult [7, 10].

The double edged sword case of maternal derived immunity (MDI) as a significant immediate defence factor for newborn piglets and its possible interference on piglet’s active immunity is a point of controversy. So far, it seems that MDI [including cell-mediated (CMI) and antibody-mediated immunity (AMI)] through colostrum and milk may interfere to some extent with the induction of AMI and CMI responses in piglets after vaccination [11–13]. Previous studies suggested that high MDI levels at the moment of vaccination could affect vaccine-induced seroconversion [5, 14–17].

The objective of this study was to assess the efficacy of a ready-to-use combination vaccine [Porcilis^®^ PCV M Hyo (MSD Animal Health, Boxmeer, The Netherlands)] against concurrent clinical M. hyo and subclinical PCV2 infection under field conditions, in the face of strong maternally derived PCV2 immunity in piglets at the time of vaccination.

## Methods

### Trial design

Farm selection was performed after pre-trial scoring of lung lesions according to the method of Goodwin and Whittlestone [18], along with PCV2 serology. Pre-trial results suggested an existing EP with typical lung lesions (cranio-ventral pulmonary consolidation) in >90% of tested lungs, along with high maternally derived PCV2 antibody titres at three weeks of piglets’ age. In addition, PCV2 antibodies were also measured after the 16th week of age and further increased until the pigs’ slaughter age (22nd week). Thus, the trial farm was considered as clinically affected with EP and subclinically affected by PCV-2. The farrow-to-finish farm was in Northern Greece with approximately 800 sows under production.

The test product is a new combination vaccine that induces immunity against PCV2 and M. hyo, and consists of Baculovirus expressed PCV2 subunits and inactivated M hyo strain J in Emunade adjuvant (Porcilis^®^ PCV M Hyo, MSD Animal Health, Boxmeer, The Netherlands). The test product evaluated in this study is registered in the European Union [19].

The efficacy evaluation of the test product was set as a placebo controlled, randomized and blinded study. Piglets were assigned individually (within litters) to the treatment groups. At admission, the animals were ear tagged as they came to hand and were allocated with the use of a computer-generated randomisation list (only known to EDT) to one of the two treatment groups, until the required number of piglets had been reached. In total, 600 three-week-old suckling piglets from three production batches (equal number of male and female) were allocated randomly to one of two groups (day 0 of the trial - December 2010). Each group consisted of 300 animals at day 0 of the study. Only clinically healthy piglets were included in the study. Diseased piglets, runts and animals with abnormalities (e.g. hernia) were excluded. On day 0 of the trial, treatment group pigs were vaccinated intramuscularly (neck area) with 2 ml of the combination vaccine and control group animals were injected in a similar way with Unisol (sterile phosphate buffered saline and sucrose). In total, 285 pigs from the treatment group and 277 control animals reached the end of the study.

**Fig. 1:**
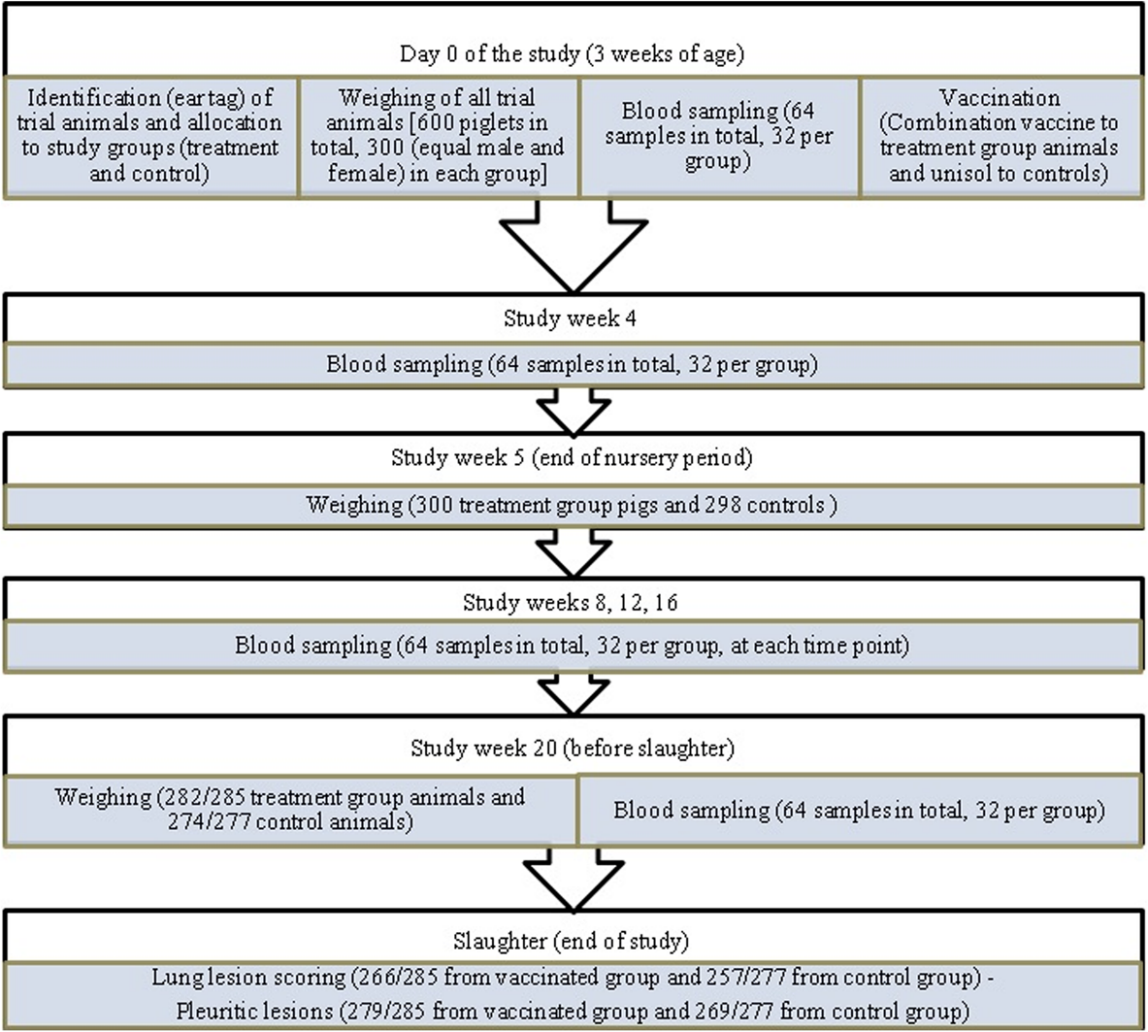
Study flow diagram

Allocation sequence was concealed until administration of the test product or Unisol to the trial animals. Enrollment, administration of products to the trial animals and assignment to each group was performed by two members (namely, P. Tassis and E. Tzika) of the research group. Animals of both groups were commingled throughout the study and they received feed and water according to the farm’s schedule. Although the test and control product were visibly different (only relevant at the time of administration), the study was blinded. After vaccination, all farm and laboratory, or other personnel involved, either carrying out observations or measurements, could not identify the vaccination status of the pigs, as it was not possible to differentiate piglets by treatment without unblinding the ear tag number. A detailed study flow diagram is presented in Fig. 1. Furthermore, the sows from which study piglets originated had been routinely vaccinated against major pathogens (PCV-2, Aujeszky’s disease virus, PRRSv, porcine parvovirus, *Erysipelothrix rhusiopathiae*, atrophic rhinitis and *Escherichia coli*).

### Recorded parameters

Each individual pig was weighed at admission, at transfer to the finishing unit (week five post-vaccination) and shortly before the first animals of each batch were transferred to the slaughterhouse [week 20 post-vaccination (end of study)]. Medication was recorded and pigs that died during the study were examined post-mortem to establish the cause of death based on gross lesions, with the exception of cases where the cause was clear and unrelated to PCV2, or M. hyo infection, or vaccination (e.g. cases of severe post-traumatic lameness). The lungs of trial animals were examined individually by the same pathologist at slaughter to score the severity of typical M. hyo lesions according to the same methodology as in the pre-trial screening [18]. The lesions of cranio-ventral pulmonary consolidation (expressed as lung lesions score - LLS) of each tested lung was the addition of the proportion of each lobe surface with signs of typical M. hyo associated inflammation (consolidated, grey to purple coloured), multiplied with the weighting factor of each lobe [20]. The LLS score ranged from 0 to 55 at maximum. Also pleuritis lesions were scored as Grade 0 - no pleuritis lesions, Grade 1 - topical adhesions (spots) and Grade 2 - larger adhesions. Due to a minor data recording error, weighing data from 282 animals (out of 285) from the treatment group and 274 control pigs (out of 277) were analyzed at slaughter. Similarly, pleuritic lesions were evaluated from 279 vaccinated and 269 control pigs. Technical issues during lung extraction at slaughter, led to the evaluation of LLS data from a total of 266 treated animals and 257 controls.

The LLS, average daily weight gain (ADWG) in the finishing period and PCV2 viraemia results, were considered primary parameters for the assessment of vaccine efficacy. Additionally, overall ADWG (from vaccination until slaughter), mortality, morbidity (referring to the number of pigs that received individual medication) and the pleuritis lesions score, were also evaluated as secondary parameters. All procedures during this clinical study were carried out according to the Guidelines on Good Clinical Practices [21] and the animals were maintained in accordance with National and European Animal Welfare Requirements [22].

### Blood sampling and serology

Thirty-two piglets per vaccination group were bled for serum samples every four weeks, starting from day 0. To monitor field infections and to assess serological response to vaccination, the sera were tested with PCV2 ELISA [23] and M. hyo ELISA (Swine HerdChek M. Hyo IDEXX) as per manufacturer instructions. The PCV2 titres were expressed as the reciprocal of the serum dilution with a calculated maximum extinction value of 50%. For calculation of the mean and standard deviation of antibody titres, samples that were negative at the lowest dilution (<4) were set at three. Similarly, samples that were positive at the highest dilution (>14) were set at 15.

The M. hyo test results were expressed as sample to positive (S/P) ratio and scored as ‘positive’, ‘inconclusive’ or ‘negative’. Based on serology, natural PCV2 infection and relative seroconversion occurred during early finishing (12–20 weeks post vaccination), thus PCV2 levels in serum were measured by qPCR [23] during that period. The qPCR results were expressed as log10 copies/μl DNA extract. If the viral load was below the detection limit of 2 log10 copies/μl DNA extract, the result was considered negative and set at zero (0). The serum samples taken on study week 20 were also tested for antibodies to Actinobacillus pleuropneumoniae with APP OMP ELISA [24], swine influenza virus (H1N1, H1N2 and H3N2) with haemagglutination inhibition tests and PRRSv with ELISA (IDEXX PRRS X3 Ab Test).

### Statistical analysis

The pig was the statistical unit and the level of significance was set at 0.05. Sample size calculation suggested that inclusion of approximately 300 piglets in each group had 80% power to detect a difference in means of 25 g a day between groups, assuming that the common standard deviation was 100 g in a two group t-test with a 0.05 two-sided significance level. The LLS were compared between the vaccination groups using mixed model analysis of variance (ANOVA) with the lung lesions log transformed before analysis. Vaccination group was included as fixed effect and the sow and production batch as random effects. The ADWG was compared between the vaccination groups using a mixed model ANOVA. Vaccination group and gender with their interaction were included as fixed effects and sow and production batch as random effects. The bodyweight at admission was included in the model as a covariate. The proportions of pigs with pleuritis lesions (absent or present), as well as mortality and morbidity were compared between groups by Cochran Mantel Haenszel method with production batch as classification variable. The mean individual qPCR results for PCV2 viraemia were compared between groups with Wilcoxon’s rank sum test.

## Results

### Weight gain

The ADWG in the vaccinated group significantly increased by 25.3 g per day during the finishing period (Mixed model ANOVA: *P* < 0.001). Moreover, ADWG during the total study period was also significantly greater by 18.0 g per day in vaccinated pigs than controls (Mixed model ANOVA: *P* < 0.001). Results of ADWG are presented in Table 1.

**Table 1:** Mean average daily weight gain (standard error of the mean) by vaccination group and period

Period	Vaccinated group (g/day)	Control group (g/day)
Nursery period	341 (5)	349 (5)
Finishing period	770^a^(8)	745^b^(8)
Overall	665^c^(6)	647^d^(6)

^a,b,c,d^Mean values with different superscripts in the same row are significantly different. Pairwise comparison (Mixed model ANOVA): **ab**: *P* < 0.0001, **cd**: *P* < 0.0001

### PCV2 viraemia

**Fig. 2:**
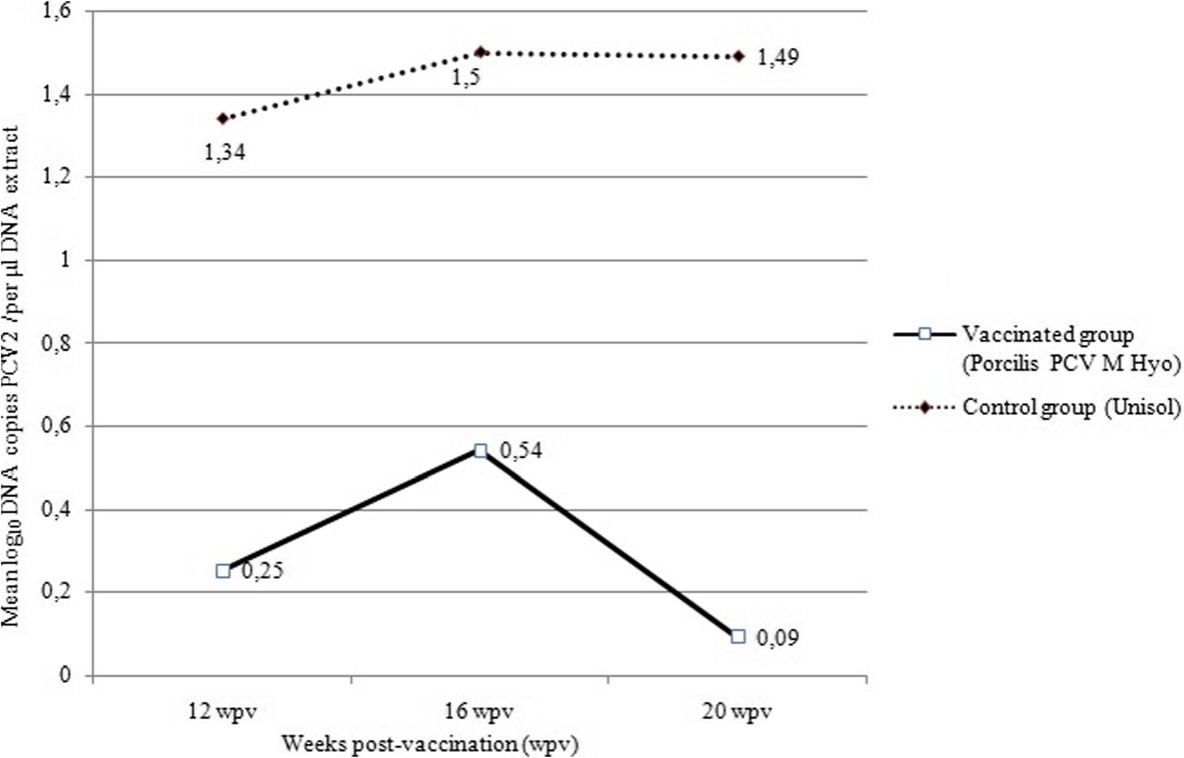
Mean log_10_ DNA copies PCV2 /per μl DNA extract, by vaccination group and weeks post-vaccination. Presented data refer only to the period when results were above the detection limits

A total of 101 samples were tested by qPCR for PCV2 viral DNA. The mean viral load was significantly (Rank Sum test: *P* = 0.0004) lower in the vaccinated pigs than the controls. The results, expressed as the mean log_10_ DNA copies per μl DNA extract, are summarised in Fig. 2. Presented data refer only to the period when results were above the detection limits.

### Lung and pleuritis lesions score

**Fig. 3:**
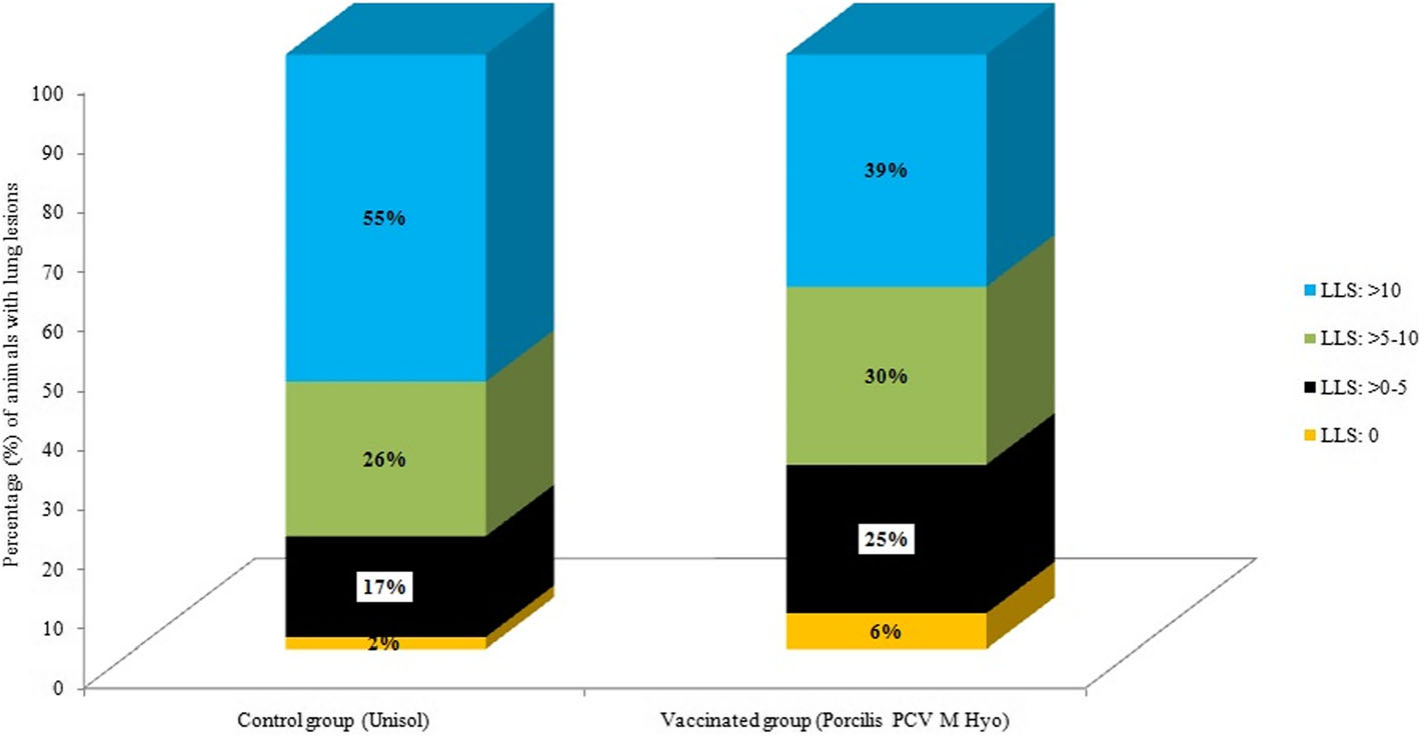
Distribution of lung lesion scores among value-range categories per group

**Fig. 4:**
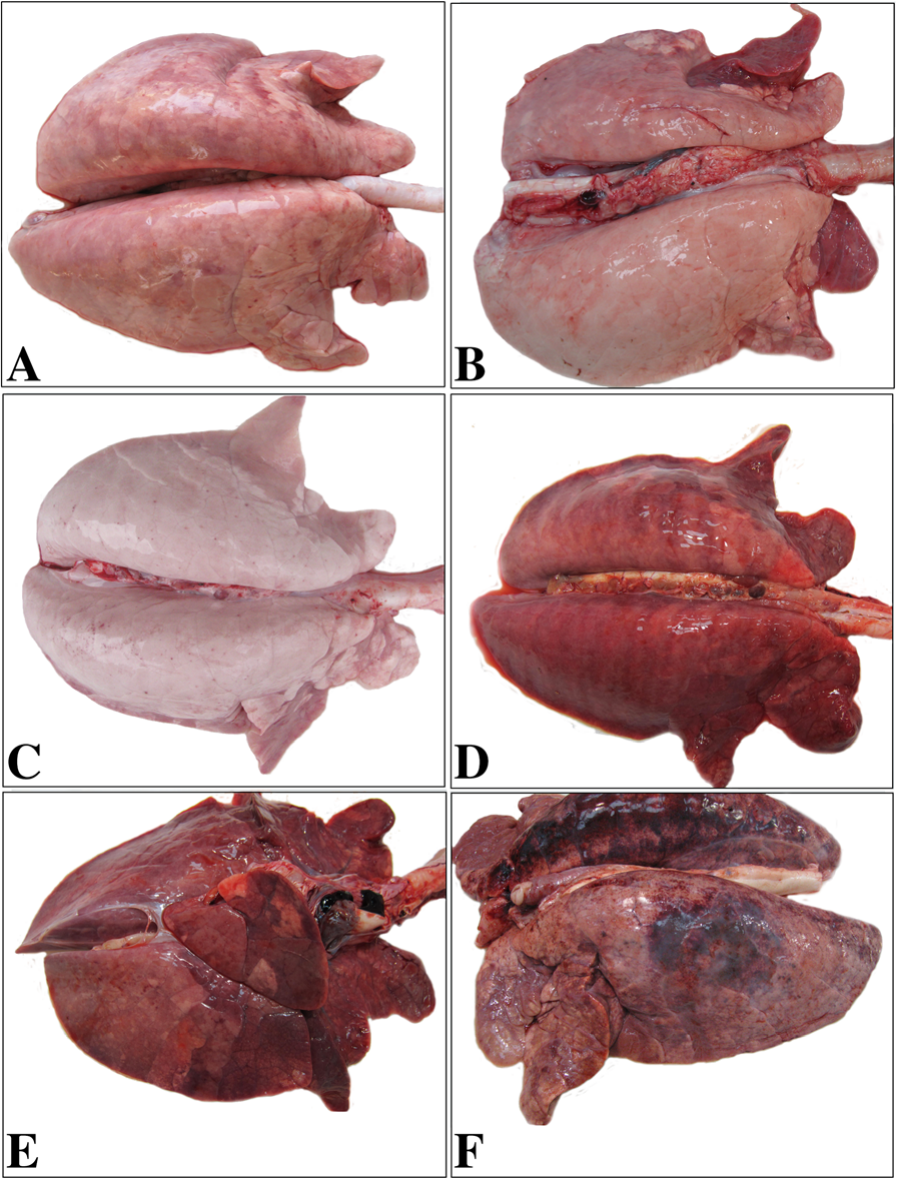
Representative findings of lung and pleuritis lesions in trial animals. Figure **a**: lung has normal appearance. Figures **b** and **c**: *purple* (**b**) and *grey* (**c**) consolidation located in cranial and middle lobes. In Fig. **c** the caudal lobes are also affected. Figure **d**: lung shows intense purple consolidation of the cranial, middle and caudal lobes. Figure **e**: in ventral view of the previous lung (**d**), lesions of bronchopneumonia are distributed in all lobes. Figure **f**: enzootic pneumonia complicated with *Actinobacillus pleuropneumoniae* infection accompanied by localized fibrous pleurisy (*left* caudal lobe). [**a** - **c**: Vaccinated group (Porcilis PCV M Hyo)] [**d** - **f**: Control group (Unisol)]

The primary parameter of LLS was significantly reduced in the vaccinated group when compared with the controls (Mixed model ANOVA *P* < 0.001). Table 2 summarizes results of LLS and pleuritis lesions among groups. The LLS was further “separated” into four categories [i.e. score = 0 (absent), score= > 0–5 (mild), score= > 5–10 (moderate), score > 10 (severe)]. The evaluation of this distribution showed that more than half of the control group cases were categorized in the fourth category, while less than 40% of the vaccinated group cases were in the same category (Fig. 3). Insignificant differences in the proportion of animals with pleuritis lesions were observed between the trial groups (*P* = 0.0759). Representative findings of lung and pleuritis lesions are presented in Fig. 4.

**Table 2:** Results of LLS and pleuritis lesions among trial groups

Lung lesions score (Mean score ± SD)		
Vaccinated group (*n* = 266)	Control group (*n* = 257)	
9.6^a^ ± 6.6	12.2^b^ ± 7.8	
Pleuritis lesions score (% of total cases examined)		
	Vaccinated group (*n* = 279)	Control group (*n* = 269)
No pleurisy lesions*	77%	70%
Topical lesions /spots	21%	25%
More extended lesions	3%	5%

^a,b,^Mean values with different superscripts in the same row are significantly different

Pairwise comparison (Mixed model ANOVA): ab: *P <* 0.0001

*Cochran Mantel Haenszel method: *P =* 0.0759

### Mortality and morbidity

In total, 38 pigs (6.3%), 15 vaccinated and 23 controls, died during the study. According to gross lesions that were reported after necropsy, respiratory disease (fibrino-necrotizing pleuropneumonia) was an important cause of death, especially in the control group (12 cases), compared with only three cases in the vaccinated group. On the other hand, gastrointestinal disease (enteritis) was the cause of death in two cases from the vaccinated group and five cases from the control group. The remaining animals died of other causes (mainly post-traumatic). Mortality between the groups was not statistically significant (*P* = 0.1799).

Fifty two vaccinated animals and 54 control piglets received individual medication, mostly for gastro-intestinal (diarrhoea) or respiratory problems. The number of treatments for respiratory signs (18) was lower than for gastro-intestinal disease (25) in the vaccinated group, while the opposite was observed in the control group (28 v 25). Morbidity between groups was not statistically significant (*P* = 0.8307).

### Serology

**Fig. 5:**
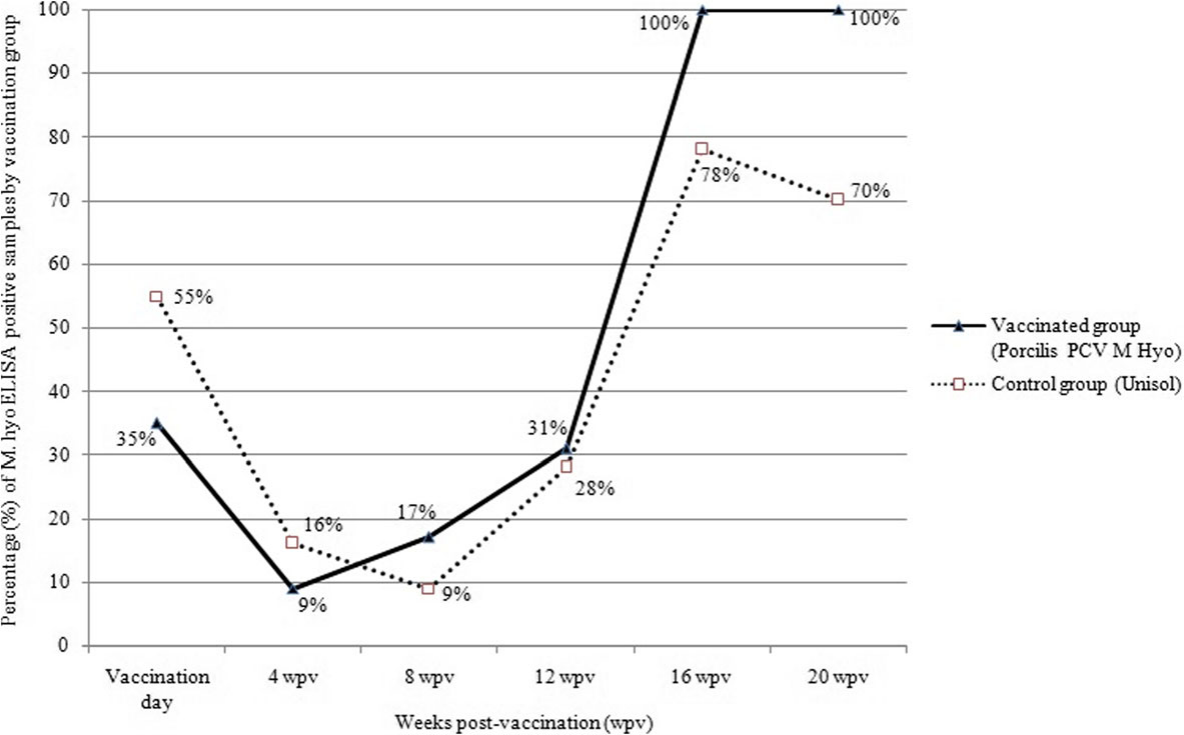
Percentage (%) of M. hyo ELISA positive samples, by vaccination group and weeks post-vaccination

**Fig. 6:**
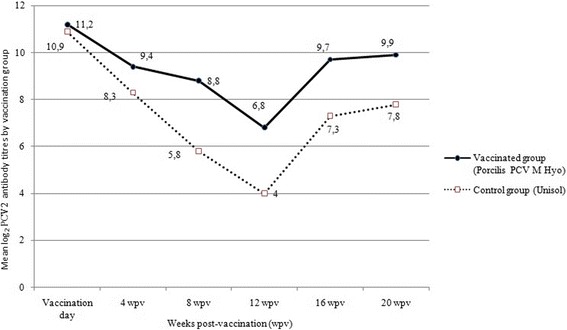
Mean log_2_ PCV2 antibody titres, by vaccination group and weeks post-vaccination

At the beginning of the study (day 0), approximately 45% of piglets were seropositive for M hyo. Towards the end of the study, the percentage of seropositive pigs increased to 100% in the vaccinated group and approximately 75% in the controls (Fig. 5). On the day of vaccination, PCV2 maternally derived antibody titre averaged 11.2 log_2_ in the vaccinated group and 10.9 log_2_ in the control group (average 11.08 log_2_ for all trial animals), which is considered high [5]. The mean PCV2 antibody titres in both treatment groups increased between 12 and 20 weeks after vaccination (Fig. 6). The maximum mean titre in the control group remained below 8 log_2_, which is considered low. At the end of the study, all pigs were seropositive for PRRSv and had moderate to high antibody titres to the outer membrane protein of *Actinobacillus pleuropneumoniae*. Low antibody titres were measured for swine influenza types H1N1 and H2N3, while the titres against type H1N2 were all below the detection limit (4 log_2_).

## Discussion

The current study demonstrates efficacy of the M hyo and PCV2 combination vaccine in 3 week old piglets in the presence of high PCV2 MDI level. Results of this study are in agreement with previous findings regarding the efficacy of the combination vaccine in the face of average PCV2 MDI levels (9.38 log_2_) [25].

Definitive diagnosis of EP is based on the demonstration of typical lung lesions [3]. The mean LLS in this study were significantly reduced in the vaccinated pigs, indicating the test product’s efficacy against M. hyo. In addition, lung lesions were also less severe in vaccinated pigs as nearly two thirds of vaccinated animals had a score < 10 compared to more than half of the controls with a score > 10. This finding further supports the vaccine’s efficacy against EP.

Moreover, during the finishing period, vaccinated pigs had a significantly better ADWG and the overall gain was 18 g per day greater than in the control pigs. The ADWG results were also in agreement with several previous studies with monovalent intramuscular M. hyo vaccination in piglets [2, 26, 27]. Since serology results suggested a subclinical PCV2 farm infection, alterations of ADWG probably can also be attributed to some extent to the control of PCV-2. The reduced viraemia after the 16th week in comparison with controls can be considered as the main factor that contributed to the PCV-2-control-related improvement of ADWG.

The M. hyo serology results on Study day 0 suggest a natural farm infection with M. hyo. Therefore, most likely maternally derived M. hyo antibodies were detected in suckling piglets on day 0, due to pathogen circulation in breeding animals. Differences in antibody titres among groups on Day 0, can be attributed to natural M. hyo infection dynamics. Differences in M. hyo circulation, and the consequent seroconversion in sows, along with the effect of serum half-life of maternally derived M. hyo antibodies, resulted in variation of their relative levels in colostrum and blood of suckling piglets. Furthermore, during the progress of the study, seroconversion and relative differences in M. hyo antibody levels between groups were observed in the last two samplings. Those differences were mainly attributed to the beneficial immune-boosting effect of vaccination. Thus, a strong immune response was observed in animals that received the test product and counteracted natural M. hyo infection with significant antibody production. On the contrary, control animals had a weaker humoral response. Similar immune response of piglets against natural M. hyo infection after administration of the combination vaccine has been previously reported [25].

Previous studies suggest that PCV2 persists in swine tissues and seroconversion has been shown in both subclinical and PCV2-SD clinically affected pigs under field conditions. In contrast, weaker immune responses have been associated with PCV2-SD clinical cases [28–30]. The trial farm was a subclinical PCV2 infected farm and a probable enhancement of PCV2 replication around the 12th week of age occurred possibly due to animal movement and/or introduction of a different PCV2 genotype [20, 31]. PCV2 serology results suggested an increased antibody response in vaccinated pigs in comparison with controls, throughout the course of the study. More important, after the time of natural infection, the humoral immune response of vaccinated animals was greater than control pigs, thus demonstrating a strong protective effect of the test product to a subclinical PCV2infection. Furthermore, qPCR results provided evidence that the mean PCV2 viral load was lower in vaccinated pigs than in controls, while viral circulation after PCV2 seroconversion was also reduced during the period when the virus was detectable.

Maternal derived immunity transfer to piglets is very important for the neonate’s immune response and survival during the first days of life. Moreover, it has been suggested that MDI is a factor that may have a significant impact on the success of vaccination under field conditions [32]. In this subclinically infected PCV2 trial farm, the level of maternally derived PCV2 antibodies on the day of vaccination was high, thus a significant negative interference with piglets immune priming could be anticipated. However, our results do not support such a negative MDI effect, considering that primary vaccine efficacy parameters such as LLS, ADWG, viraemia were all significantly improved/promoted in the vaccinated versus control animals.

## Conclusions

According to the results of the present field study, vaccination with a ready to use PCV2/M hyo combination vaccine of three-week-old piglets with high MDI was effective against concurrent clinical M. hyo and subclinical PCV-2 infection based on reduced LLS severity and PCV2 viraemia, as well as improved ADWG.

## Abbreviations

ADWG: Average daily weight gain
AMI: Antibody-mediated immunity
CMI: Cell-mediated immunity
EP: Enzootic pneumonia
LLS: lung lesion score
M. hyo: *Mycoplasma hyopneumoniae*
MDI: Maternal derived immunity
PCV2: Porcine circovirus type 2
PCV2-SD: PCV2 - Systemic disease
PCVD: Porcine circovirus diseases
PRRSv: Porcine reproductive and respiratory syndrome virus
SD: Standard deviation
